# Anti-viral Effect of *Bifidobacterium adolescentis* against Noroviruses

**DOI:** 10.3389/fmicb.2016.00864

**Published:** 2016-06-08

**Authors:** Dan Li, Adrien Breiman, Jacques le Pendu, Mieke Uyttendaele

**Affiliations:** ^1^Laboratory of Food Microbiology and Food Preservation, Faculty of Bioscience Engineering, Ghent UniversityGhent, Belgium; ^2^UMR 892, Institut National de la Santé et de la Recherche MédicaleNantes, France; ^3^UMR 6299, Centre National de la Recherche ScientifiqueNantes, France; ^4^Faculty of Medicine, University of NantesNantes, France; ^5^Nantes University HospitalNantes, France

**Keywords:** *Norovirus*, *Bifidobacterium adolescentis*, probiotics, mechanisms

## Abstract

This study aims to investigate the effect of *Bifidobacterium adolescentis* against noroviruses (NoVs). Murine norovirus-1 (MNV-1) used as a surrogate was detected by plaque assay and RT-qPCR. Human NoV virus like particles (VLPs) were detected by cell-binding assay. It was shown that the presence of *B. adolescentis* could inhibit the multiplication of MNV-1 on RAW 264.7 cells within 48 h of co-incubation period at 37°C. This inhibition did not occur at the viral binding stage, as no difference was observed in MNV-1 genomic copies collected from washed RAW 264.7 cells without and with *B. adolescentis* after co-incubation for 1 h at room temperature. Meanwhile, the presence of *B. adolescentis* decreased the binding of human NoV GI.1 VLPs to both Caco-2 cells and HT-29 cells, while no reduction was induced for the binding of human NoV GII.4 VLPs to Caco-2 cells.

## Introduction

Probiotics are defined as “living micro-organisms, which upon ingestion in certain numbers, exert health benefits beyond inherent basic nutrition.” Most probiotic microorganisms belong to lactic acid bacteria (LAB), such as *Lactobacillus* sp., *Bifidobacterium* sp., and *Enterococcus* sp (Ljungh and Wadström, 2006). Probiotics may benefit the human and animal host directly, by preventing the infection and combating the causative agent of the intestinal disorder, or indirectly, by balancing the disrupted equilibrium of the enteric flora and augmenting the host’s immune responses (Maragkoudakis et al., 2010; Sanders et al., 2013). Clinical evidence has reported that feeding of probiotics can prevent effectively for diarrhea and shedding of rotavirus (Saavedra et al., 1994; Van Niel et al., 2002; Sazawal et al., 2006; Grandy et al., 2010). Accordingly, with the use of animal models, multiple probiotic strains have shown anti-rotavirus effect (Muñoz et al., 2011; Kandasamy et al., 2014; Mao et al., 2016). In addition, *in vitro* studies have demonstrated that probiotics may have antiviral activity against rotavirus (Maragkoudakis et al., 2010; Muñoz et al., 2011), coxsackievirus (Kim et al., 2014), hepatitis C virus (El-Adawi et al., 2015), as well as noroviruses (NoVs; Aboubakr et al., 2014; Rubio-del-Campo et al., 2014).

Noroviruses, one genera of the *Caliciviridae* family, were reported as the cause of between 73% to greater than 95% of global epidemic nonbacterial gastroenteritis outbreaks and approximately half of all gastroenteritis outbreaks (Atmar and Estes, 2006). Although NoV infections are generally mild, it may require hospital care and can be associated with mortality in elderly, chronically ill or immune-compromised patients. Taking into consideration their widespreadness, NoVs are causing heavy disease burdens associated with large economic losses (Van Beek et al., 2013). Despite recent progress, several key challenges remain in assessing the efficacy of vaccines and antiviral drugs for human NoV infection, such as the lack of a robust cell culture system or animal model limits direct study of these viruses, and the extreme genetic heterogeneity among strains (Karst et al., 2014). Therefore, it is of high interest to investigate further if probiotics can be employed for NoV control and treatment.

In this study, due to the non-cultivability of human NoVs, murine norovirus-1 (MNV-1, a very commonly used human NoV surrogate, Kniel, 2014), and human NoV virus like particles (VLPs) were used to study the effect of *Bifidobacterium adolescentis* against NoVs.

## Materials and Methods

### Bacteria

*Bifidobacterium adolescentis* (LMG10502, biological origin: adult intestines) was obtained from Belgian Coordinated Collection of Microorganisms (BCCM/LMG). *B. adolescentis* was cultured in tryptone soya broth (TSB, Oxoid, Thermo) at 37°C. The anaerobic atmosphere was generated with the use of ANAEROGEN^TM^ COMPACT (Oxoid, Thermo).

Before each experiment, *B. adolescentis* were cultured for 48 h, normalized to an OD_570_ of 0.4, and washed twice with phosphate buffered saline (PBS, pH 7.4).

### Cell Lines, Virus, and Virus-Like Particles (VLPs)

Cells of the murine macrophage cell line RAW 264.7 (ATCC TIB-71; kindly provided by Prof. H. W. Virgin, Washington University School of Medicine, St. Louis, MO, USA) were maintained in complete DMEM medium and grown at 37°C under a 5% CO_2_ atmosphere. Complete DMEM consisted of Dulbecco’s modified Eagle’s medium (DMEM; Lonza, Walkersville, MD, USA) containing 10% low-endotoxin fetal bovine serum (HyClone, Logan, UT, USA), 100 U/ml penicillin, 100 μg/ml streptomycin (Lonza), 10 mM HEPES (Lonza), and 2 mM L-glutamine (Lonza).

RAW 264.7 cells were infected with MNV-1.CW1 and passaged seven times at a multiplicity of infection (MOI) of 0.05 (MNV-1:cells) for 2 days. After two freeze-thaw cycles, low speed centrifugation was used to remove cellular debris from the virus suspension, as described by Wobus et al. (2004). The lysate containing suspended MNV-1 was stored in aliquots at –75°C.

Cell line Caco-2 (ECACC 86010202) was cultured in Eagle’s minimum essential medium with Earle’s salts (EMEM; Lonza) supplemented with 10% low-endotoxin fetal bovine serum (HyClone), 100 U/ml penicillin, 100 μg/ml streptomycin (Lonza), and 2 mM L-glutamine (Lonza) and grown at 37°C under a 5% CO_2_ atmosphere. HT-29 (ATCC HTB-38) cells were cultured in Dulbecco’s modified Eagle’s medium (DMEM; GIBCO^®^, Life Technologies.) supplemented with 10% low-endotoxin fetal bovine serum (GIBCO^®^, Life Technologies), 100 U/ml penicillin, 100 μg/ml streptomycin (GIBCO^®^, Life Technologies), and 2 mM L-glutamine (GIBCO^®^, Life Technologies) and grown at 37°C under a 5% CO_2_ atmosphere.

Virus-like particles of human NoV GI.1 (Norwalk virus) and GII.4 (Dijon 1996) were generated using a previously described method (Jiang et al., 1992). Recombinant baculoviruses containing the VP1 protein from NoV GI.1 and GII.4 were generated, and VLPs were produced by infection of Hi5 insect cells. VLPs were released from the infected Hi5 cells by three rounds of freeze-thawing and then clarified by removal of cellular debris (6,000 × g for 30 min) and baculovirus (14,000 × g for 30 min). The VLPs were partially purified through a 30% (wt/vol) sucrose cushion in TNC buffer (50 mM Tris-HCl, pH 7.4, 150 mM NaCl, 10 mM CaCl_2_) containing the protease inhibitor leupeptin at 150,000 × g for 2 h. The pelleted VLPs were resuspended in TNC and further purified by isopynic centrifugation in cesium chloride (150,000 × *g*; 18 h). The resultant VLP bands were collected by puncture, and the solution containing VLPs was dialyzed against PBS prior to quantification by bicinchoninic acid (BCA) protein assay (Thermo Scientific) and stored at –80°C.

### Cytotoxicity Test-Neutral Red Assay

The cytotoxicity of bacteria on RAW 264.7 cells was measured by a neutral red assay based on the description by Fotakis and Timbrell (2006) with a few modifications. The RAW 264.7 cells were seeded into 96-well plates at a density of 10^5^ viable cells per well. On the following day, the RAW 264.7 cells were washed with PBS to remove the antibiotics, the *B. adolescentis* re-suspended in DMEM (Lonza) were added onto the cells (50 μl per well) and incubated for 1 h at room temperature. Afterward the inoculum was aspirated and fresh complete DMEM without antibiotics was added (100 μl per well). Plates were incubated at 37°C and 5% CO_2_.

After 2 days incubation, the medium of the cells was changed to neutral red dye (Sigma–Aldrich, St. Louis, MO, USA, 100 μg/ml) dissolved in DMEM (100 μl per well) and incubated for another 2 h at 37°C and 5% CO_2_. Cells were then washed with PBS and the addition of elution medium (EtOH/AcCOOH, 50%/1%, 100 μl per well) followed by gentle shaking for 10 min so that complete dissolution was achieved. The optical density was read at 540 nm (OD_540_).

### Viral Multiplication Inhibition Test on MNV-1 Detected by Plaque Assay

The RAW 264.7 cells were seeded into six-well plates at a density of 2 × 10^6^ viable cells per well. On the following day, the *B. adolescentis* re-suspended in DMEM (Lonza) was used to make ten-fold dilutions from MNV-1 lysate. The RAW 264.7 cells were washed with PBS to remove the antibiotics, the mixture of bacteria and MNV-1 was added onto the cells (500 μl per well, two wells per sample). Plates were incubated for 1 h at room temperature and manually rocked every 15 min before aspirating the inoculum and overlaying the cells with 1.5% SeaPlaque agarose (Cambrex, Rockland, ME, USA) in MEM (Lonza) supplemented with 10% low-endotoxin fetal bovine serum, 1% HEPES, 1% penicillin/streptomycin, and 2% glutamine (complete MEM) per well. Plates were incubated at 37°C and 5% CO_2_ for 2 days. To visualize plaques, cells were stained with 1.5% SeaKem agarose in complete MEM containing 1% neutral red (Sigma–Aldrich) per well for 6 h.

Plaque sizes were shown to be associated with the virulence in multiple studies (Takemoto, 1966; Lipton, 1980). In this study, from photos taken from each group with the same format and size, the diameters of plaques were measured and recorded by the use of ImageJ (National Institutes of Health, Bethesda, MD, USA).

### Viral Binding Inhibition Test on MNV-1 Detected by RT-qPCR

The RAW 264.7 cells were seeded into 96-well plates at a density of 10^5^ viable cells per well. On the following day, the *B. adolescentis* was re-suspended in MNV-1 dilutions with DMEM (50 μl per sample, with 5.8 ± 0.1 log MNV-1 genomic copies per sample). The RAW 264.7 cells were washed with PBS to remove the antibiotics, the mixture of bacteria and MNV-1 were added onto the cells (50 μl per well) and incubated for 1 h at room temperature. Afterward the inoculum was aspirated and the cells were washed by PBS for three times.

The RNAs of washed cells were extracted by using the RNeasy Mini kit (Qiagen, Hilden, Germany). For each sample the 100 μl PBS was firstly mixed with 350 μl lysis buffer, and the mixture was pipetted back onto the cells in the 96-well plates in order to lyse the cells. The following procedures were performed according to the RNA Cleanup protocol and the RNA were stored at –75°C.

The RT-qPCR of MNV-1 was performed by the use of RNA UltraSense^TM^ One-Step Quantitative RT-PCR System (Life technologies). The primers and probe sequence of MNV-1 were previously shown by Baert et al. (2008). Twenty micro liter of reaction mixture [200 nM each primer, 200 nM probe, 50 nM ROX (Life technologies)] was added to 5 μl of RNA. The RT-qPCR assays were performed in an ABI 7300 system (Applied Biosystems). The amplification profile consisted of 50°C for 15 min, 95°C for 2 min and 45 cycles of 95°C for 15 s and 60°C for 30 s.

An absolute quantification of MNV-1 genomic copies was performed as described by Baert et al. (2008). To obtain representative positive control standards, the plasmid p20.3 containing a full-length cDNA clone of MNV-1.CW1 (Baert et al., 2008) was used for the quantifications. Ten-fold serial dilutions ranging from 10^6^ to 10 copies of plasmids per reaction were used to prepare the standard curves.

### Viral Binding Inhibition Test on Human NoV VLPs Detected by Fluorescence Measurement

The HT-29 cells were seeded into 96-well plates at a density of 10^5^ viable cells per well. On the following day, the *B. adolescentis* was re-suspended in NoV GI.1 VLP suspension [5 μg/ml, in PBS-0.1% bovine serum albumin (BSA)]. The mixture of bacteria and VLPs was added onto the cells (50 μl per well) and incubated for 1 h at room temperature. After washing with PBS for three times, the cells were fixed with 4% paraformaldehyde, and stained by anti-VLP rabbit polyclonal antibodies (lp130 for GI.1, diluted in PBS-0.1% BSA, 1-h incubation at 37°C) followed by Alexa Fluor^®^ 488 Goat Anti-Rabbit IgG (H+L) antibody (Life Technologies, diluted in PBS-0.1% BSA, 1-h incubation at 37°C). Three times washing by PBS was always performed after each step. The fluorescence was read by a fluorimeter (Fluoroskan, Thermo Scientific; Ex/Em = 490/525 nm) in arbitrary unit (a.u.).

The binding test of human NoV GI.1 and GII.4 VLPs (anti-VLP rabbit polyclonal antibodies lp130 for GI.1 and lp132 for GII.4) on Caco-2 cell were similar with the procedures above except that the Caco-2 cells were incubated for 21 days post confluency to be used as differentiated cells.

The binding of NoV GI.1 VLPs to HT-29 cells was also observed with a fluorescence microscope. The HT-29 cells were seeded into eight-well Nunc^®^ Lab-Tek^®^ Chamber Slide System (Sigma–Aldrich) at a density of 10^5^ viable cells per well. On the following day, the binding and staining steps were the same as described above. After the last washing step (from the secondary antibody incubation), the upper structure was removed from the bottom glass slide. The stained cells were mounted on slides with Vectashield^®^ (Vector laboratories, Burlingame, CA, USA). The sealed slides were observed under a Zeiss Axiovert 200 fluorescence microscope.

### Data Analysis

Each result was presented as the mean value of three independent replicates with the standard deviation. Statistical analyses were performed by Mann–Whitney *U* test with SPSS 22 for Windows (SPSS, Inc., Chicago, IL, USA). Significant differences were considered when *P* was <0.05.

## Results

### Effect of *B. adolescentis* on MNV-1 Infectivity

First of all, after 48 h incubation, the cell viability determined by neutral red assay showed no significant reduction after incubation of the RAW 264.7 cells with *B. adolescentis* (OD_540_ 1.1 ± 0.1–1.1 ± 0.2, *P* > 0.05).

**FIGURE 1:**
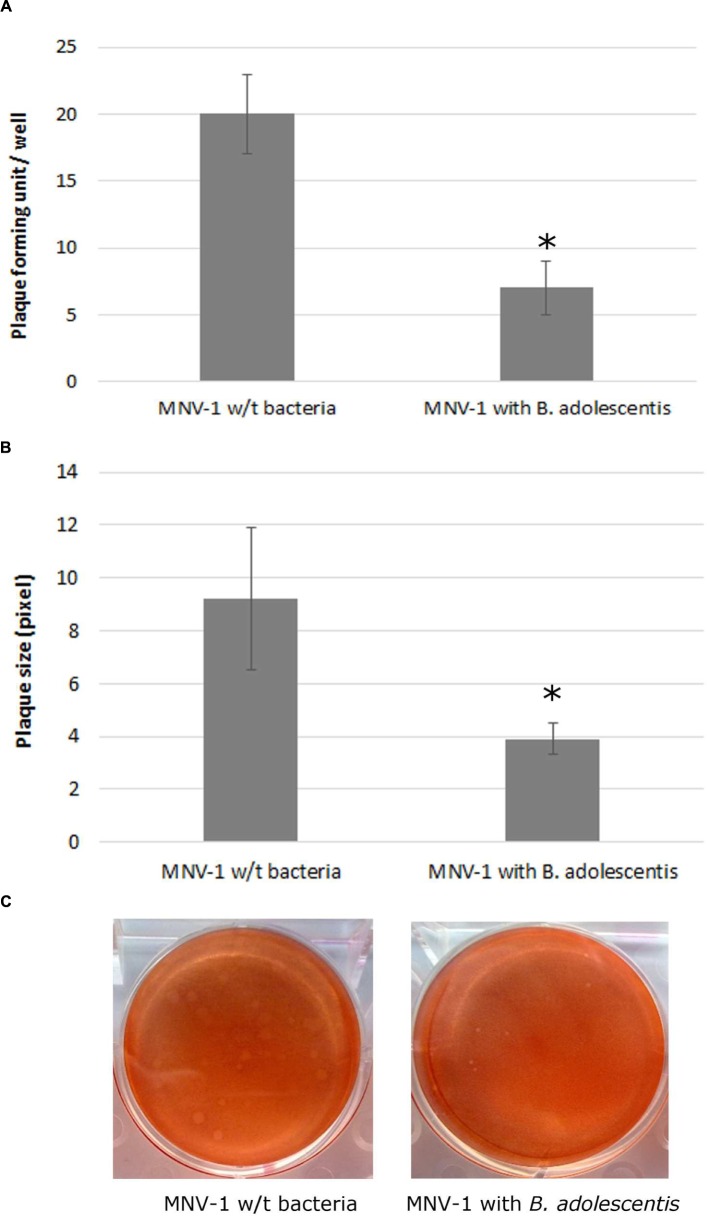
**MNV-1 multiplication inhibition by *B. adolescentis* determined by plaque assay.** **(A)** Plaque forming units (PFU) from cell-culture wells with and without *B. adolescentis*. Each data point is an average of three independent tests, and each error bar represents the data range. ^∗^*P* < 0.05. **(B)** Plaque diameters from cell-culture wells with and without *B. adolescentis*. Each data point is an average of 15 measurements, and each error bar represents the data range. ^∗^*P* < 0.05. **(C)** Plaque appearances from cell-culture wells with and without *B. adolescentis*.

MNV-1 lysate was diluted to a concentration that can form countable plaques on the six-well plates, and was seeded onto the cells with or without *B. adolescentis*. The plaque assay showed that compared with the negative control (MNV-1 on RAW 264.7 cells without bacteria), the MNV-1 plaque forming units (PFU) from cell-culture wells in the presence of *B. adolescentis* were decreased significantly from 20 ± 3–7 ± 2 PFU/well (**Figure 1A**, *P* < 0.05).

It was also noticed that the sizes of the plaques from cells incubated with *B. adolescentis* were smaller than the negative control (examples shown in **Figure 1C**). The average sizes of the plaques measured by ImageJ showed that compared with the negative control (MNV-1 on RAW 264.7 cells without bacteria), the plaque diameters from cell-culture wells in the presence of *B. adolescentis* were decreased significantly from 9.2 ± 2.7–3.9 ± 0.6 pixel (**Figure 1B**, *P* < 0.05). These results indicate that the effect of *B. adolescentis* on MNV-1 may occur mainly in the viral replication phase instead of showing direct virucidal effect on the MNV-1 or preventing the viruses from binding to the cells.

Therefore lastly, a viral binding inhibition test was performed and indeed no significant difference of MNV-1 genomic copies collected from RAW 264.7 cells between the two groups with (5.77 ± 0.03 log-genomic copies/ml) and without *B. adolescentis* (5.74 ± 0.01 log-genomic copies/ml) was observed (*P* > 0.05).

### Effect of *B. adolescentis* on Human NoV VLPs Cell-Binding Ability

It has been previously reported that both VLPs of human NoV GI.1 and GII.4 could bind to Caco-2 cells (Tamura et al., 2004) and VLPs of human NoV GI.1 could bind to HT-29 cells (Rubio-del-Campo et al., 2014).

**FIGURE 2:**
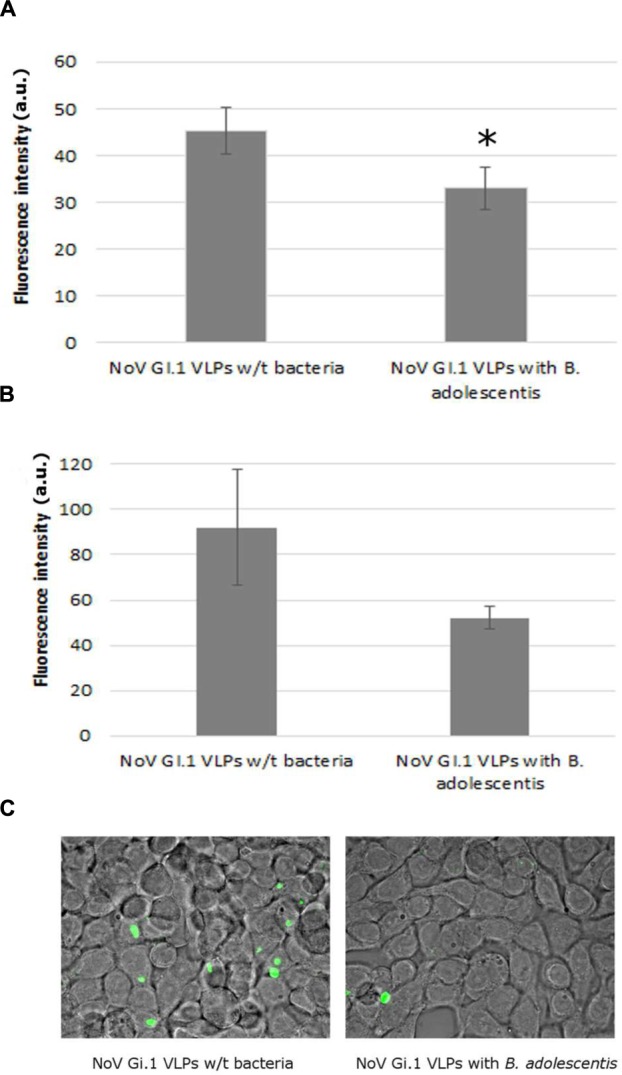
**Human NoV GI.1 VLPs binding inhibition by *B. adolescents*.** **(A)** Fluorescence measurement in arbitrary unit (a.u.) of human NoV GI.1 VLPs bound to Caco-2 cells. Each data point is an average of three independent tests, and each error bar represents the data range. ^∗^*P* < 0.05. **(B)** Fluorescence measurement in arbitrary unit (a.u.) of human NoV GI.1 VLPs bound to HT-29 cells. Each data point is an average of three independent tests, and each error bar represents the data range. **(C)** Fluorescence microscope observation of human NoV GI.1 VLPs bound to HT-29 cells.

The presence of *B. adolescentis* decreased the binding of human NoV GI.1 VLPs to Caco-2 cells, measured by fluorescence intensity, significantly from 45.2 ± 5.0 (negative control without bacteria) to 33.0 ± 4.6 a.u. (incubation with *B. adolescentis*; **Figure 2A**, *P* < 0.05).

The fluorescence intensity of human NoV GI.1 VLPs on HT-29 cells also decreased, although not significantly, from 92.0 ± 25.0 to 52.0 ± 5.0 a.u. by the incubation of *B. adolescentis* (**Figure 2B**, *P* = 0.05). A visual example detected by fluorescence microscopy was shown in **Figure 2C**.

As for human NoV GII.4 VLPs, no significant reduction was observed for the binding of the VLPs to Caco-2 cells with the incubation of *B. adolescentis* (19.0 ± 1.7 to 17.7 ± 0.6 a.u., *P* > 0.05).

## Discussion

Currently, the research on the effect of probiotics on NoVs are still preliminary. On one hand, due to the non-cultivability of human NoVs, the *in vitro* studies were conducted with the use of surrogate viruses [e.g., feline calicivirus (FCV), Aboubakr et al., 2014; MNV-1 and Tulane virus (TV), Shearer et al., 2014] and artificially synthesized particles partially mimicking the viral structures (*P*-particles, Rubio-del-Campo et al., 2014), which may introduce gaps from the authentic NoV reactions. On the other hand, there are an insufficient number of in-depth studies on this topic before a general conclusion can be drawn. For instance, it was reported that the fermentation of *Donchimi* and oysters could effectively reduce the infectivity of FCV and MNV-1 (Lee et al., 2012; Seo et al., 2014). The population of LAB increased largely during the fermentation, however, it was neither clear on the role that the LAB played nor the associated mechanisms (Lee et al., 2012; Seo et al., 2014). For another instance, high infectivity reduction (6 –7-log reduction) was observed for FCV following co-incubation with *Lactococcus lactis* (both bacterial medium filtrate and cells suspension, Aboubakr et al., 2014). However, the cell-free supernatant of a commercial probiotic mixture of *Lactobacillus acidophilus, Lactobacillus rhamnosus, Bifidobacterium bifidum, Lactobacillus salivarius, and Streptococcus thermophiles* indicated no reduction for the infectivity of MNV and TV (Shearer et al., 2014). The reasons causing the inconsistence of the studies can be the differences between both of the studied viruses and bacteria strains, as well as the experimental settings such as time/sequence of addition, comparative ratio of viruses and bacteria, incubation conditions (time, atmosphere and temperature) and matrices, etc.

In this study, MNV-1 was employed as the surrogate as it was more persistent than FCV in the fermentation of both *Donchimi* and oysters (Lee et al., 2012; Seo et al., 2014). Human NoV VLPs from GI.1 and GII.4 were used as they could mimic the binding capacity of two representative NoV genotypes. *B. adolescentis* is a recognized probiotics (Lee et al., 1993; Kim et al., 2008) and has interested dairy manufacturers in producing “therapeutic fermented milk products” (Arunachalam, 1999; Puniya, 2015). Also it was reported recently to exhibit antiviral activity against Coxsackievirus (Kim et al., 2014). The bacterial cells were washed with PBS before being added to the viruses and cells in order to avoid any direct virucidal effect of the bacterial metabolites in the culture medium, such as organic acids, diacetyl, and bacteriocins. The bacteria and viruses (MNV-1 and human NoV VLPs) were co-incubated with the cells as this strategy was shown to be more effective than pre-treatment of cells, pre-treatment of viruses (Aboubakr et al., 2014; Rubio-del-Campo et al., 2014), or post-treatment of cells attached to viruses (Rubio-del-Campo et al., 2014).

It was demonstrated in this study that the presence of *B. adolescentis* could inhibit the multiplication of MNV-1 on RAW 264.7 cells. Meanwhile, it was shown that this inhibition effect did not occur on the binding stage of MNV-1 to RAW 264.7 cells. However, in contrast, the presence of *B. adolescentis* did decrease the binding of human NoV GI.1 VLPs to both Caco-2 cells and HT-29 cells. Although based on different models, these results indicate that the effects of *B. adolescentis* on different viruses might vary based on different mechanisms.

It was reported previously that NoV P-particles could bind to a series of probiotics with varied binding ability, which was postulated as one of the antiviral mechanisms of the probiotics (Rubio-del-Campo et al., 2014). However, based on a recent study of our group (Li et al., 2015), the *B. adolescentis* strain used in this study does not express histo-blood group antigen (HBGA) and could not bind to NoV VLPs of either GI.1 or GII.4. This result rules out the possibility of direct competition of *B. adolescentis* and NoV GI.1VLPs from binding to Caco-2 or HT-29 cells. Instead, as we and others have described a few bacterial lectins binding to HBGAs (Gilboa-Garber et al., 2011; Audfray et al., 2012) and Zhong et al. (2004) have reported that the adhesin of *B. adolescentis* 1027 could inhibit the adhesion of *Escherichia coli* to intestinal epithelial cell line Lovo, we assume that certain lectins secreted by *B. adolescentis* could bind to the HBGAs on the intestinal cell lines and compete with the binding of NoV GI.1VLPs.

As the binding of human NoV GI.1 VLPs to both Caco-2 cells and HT-29 cells was decreased in the presence of *B. adolescentis*, it is reasonable to assume that the viral multiplication of human NoV GI.1, if an *in vitro* model could be established, should also be decreased in the presence of *B. adolescentis.* On the other hand, although no reduction of the binding of human NoV GII.4 VLPs to Caco-2 cells was observed in the presence of *B. adolescentis*, the possibility cannot be ruled out that *B. adolescentis* may reduce the viral multiplication of human NoV GII.4 if an *in vitro* model could be established, or help combat NoVs *in vivo*. The differences observed between the two strains are not surprising as it is well known that the chemical structures of the receptor binding interfaces are different between NoV GI.1 and GII.4 (Tan and Jiang, 2011). However, the exact molecular mechanisms casing the influence of *B. adolescentis* on human NoV VLPs binding to intestinal cell lines still need further investigation.

## Conclusion

This study demonstrated the antiviral effect of *B. adolescentis* against NoVs. Due to the lack of models to study the infection of genuine human NoVs, the results were generated from studying surrogates detected by different methods (infection and binding of MNV-1 on murine macrophage cell line RAW 264.7and binding of human NoV VLPs on human intestinal epithelial cell lines Caco-2 and HT-29). Since the effects of *B. adolescentis* on MNV-1 and human NoV VLPs as well as their mechanisms were indicated to be different, this study shows the importance of establishing multiplication model for human NoVs in the future. In addition, more probiotic strains will be tested before a general conclusion can be drawn on the antiviral effect of probiotics on NoV infection.

## Author Contributions

DL performed most of the experiments and composed the manuscript. AB supplied technical support for the experiments, especially for the fluorescence microscope as well as improvement of the manuscript. JP and MU gave strategic guidance for the work and suggestions to improve the manuscript.

## Conflict of Interest Statement

The authors declare that the research was conducted in the absence of any commercial or financial relationships that could be construed as a potential conflict of interest.
